# Caveolin-1 suppresses tumor formation through the inhibition of the unfolded protein response

**DOI:** 10.1038/s41419-020-02792-4

**Published:** 2020-08-03

**Authors:** María I. Díaz, Paula Díaz, Jimena Castillo Bennett, Hery Urra, Rina Ortiz, Pamela Contreras Orellana, Claudio Hetz, Andrew F. G. Quest

**Affiliations:** 1 Laboratory of Cellular Communication, Center for studies on Exercise, Metabolism and Cancer (CEMC), Faculty of Medicine, University of Chile, Santiago, Chile; 2 Program of Cellular and Molecular Biology, Institute of Biomedical Sciences (ICBM), University of Chile, Santiago, Chile; 3 Advanced Center for Chronic Diseases (ACCDiS), Santiago, Chile; 4 Biomedical Neuroscience Institute (BNI), Faculty of Medicine, University of Chile, Santiago, Chile; 5 FONDAP Center for Geroscience, Brain Health and Metabolism (GERO), Santiago, Chile; 6 Laboratory of Proteostasis Control and Biomedicine, Institute of Biomedical Sciences, Faculty of Medicine, University of Chile, Santiago, Chile; 7 Buck Institute for Research on Aging, Novato, CA 94945 USA

**Keywords:** Tumour-suppressor proteins, Mechanisms of disease, Endoplasmic reticulum

## Abstract

Caveolin-1 (CAV1), is a broadly expressed, membrane-associated scaffolding protein that acts both, as a tumor suppressor and a promoter of metastasis, depending on the type of cancer and stage. CAV1 is downregulated in human tumors, tumor cell lines and oncogene-transformed cells. The tumor suppressor activity of CAV1 is generally associated with its presence at the plasma membrane, where it participates, together with cavins, in the formation of caveolae and also has been suggested to interact with and inhibit a wide variety of proteins through interactions mediated by the scaffolding domain. However, a pool of CAV1 is also located at the endoplasmic reticulum (ER), modulating the secretory pathway in a manner dependent on serine-80 (S80) phosphorylation. In melanoma cells, CAV1 expression suppresses tumor formation, but the protein is largely absent from the plasma membrane and does not form caveolae. Perturbations to the function of the ER are emerging as a central driver of cancer, highlighting the activation of the unfolded protein response (UPR), a central pathway involved in stress mitigation. Here we provide evidence indicating that the expression of CAV1 represses the activation of the UPR in vitro and in solid tumors, reflected in the attenuation of PERK and IRE1α signaling. These effects correlated with increased susceptibility of cells to ER stress and hypoxia. Interestingly, the tumor suppressor activity of CAV1 was abrogated by site-directed mutagenesis of S80, correlating with a reduced ability to repress the UPR. We conclude that the tumor suppression by CAV1 involves the attenuation of the UPR, and identified S80 as essential in this context. This suggests that intracellular CAV1 regulates cancer through alternative signaling outputs.

## Introduction

Cancer is a multi-factorial disease that involves the loss of a cell’s ability to respond to cues provided by the microenvironment. Depending on whether these changes involve gain-of-function or loss-of-function, the respective molecules are classified as either oncogenes or tumor suppressors^1,2^. Caveolin-1 (CAV1) is a membrane-associated protein that is highly enriched in specialized, 50–100 nm omega-shaped invaginations of the plasma membrane, known as caveolae^3^. Besides caveolae biogenesis, CAV1 is implicated in cholesterol transport, endocytosis, cell signaling and the development of different diseases beyond cancer^3–6^.

In cancer, CAV1 operates both, as a tumor suppressor gene and a promoter of metastasis, depending on the type of cancer and stage^7,8^. On one hand, CAV1 promotes the migration of tumor-derived cancer cells by mechanisms that appear to depend on the cellular context^9^. As well, in later stages of cancer, the expression of CAV1 increases and favors the development of cellular characteristics related to enhanced malignancy, promoting tumor cell progression, invasion and metastasis^7,10^.

On the other hand, CAV1 expression is reduced in several human cancers, including lung^11^, mammary^12^, colon^13,14^, and ovarian carcinomas, as well as sarcomas^15^. In addition, the re-expression of CAV1 often results in reversal of the characteristics associated with the transformed phenotype^13–15^. Consistent with these results, lung hyperplasia, predisposition to mammary, as well as carcinogen-induced skin hyperplasia and tumor formation are observed in CAV1 knock-out mice^16–18^. Overall, these results indicate that CAV1 displays properties characteristic of a tumor suppressor in a variety of cellular models.

At the molecular level, CAV1 is viewed as a negative regulator of various pathways relevant to cell proliferation and survival due to inhibitory interactions with cancer-relevant signaling proteins. These interactions depend on the CAV1 scaffolding domain (CSD: amino acids 82–101), a modular sequence located adjacent to the hydrophobic domain in the amino-terminal region of CAV1, and the scaffolding domain binding domain (CBD), located in target proteins^10,19,20^. Moreover, CSD/CBD domain interactions connect CAV1 to cell death, either directly by favoring the induction of apoptosis or indirectly via sensitization to cell death^10,21–23^. Previous results from our group linked the tumor suppressor role of CAV1 to its ability to suppress β-catenin/Tcf-Lef-dependent transcription of genes including survivin and COX2 in an E-cadherin-dependent manner^24,25^.

Although most of CAV1 functions mentioned above are generally related to the presence of CAV1 at the plasma membrane, we previously demonstrated using murine and human melanoma models that CAV1 suppresses tumor formation despite having a reduced expression at the plasma membrane^26^. Indeed, several studies have shown that CAV1 also locates to various subcellular compartments including the Golgi apparatus, endosomes, mitochondria, the nucleus and the endoplasmic reticulum (ER)^27–29^. Early studies suggested that CAV1 is implicated in the regulation of secretory processes at the ER by a poorly described regulatory mechanism that involves the amino acid residue serine-80^30^. Recently, the presence of CAV1 in ER-mitochondria contact sites was also described^31^, suggesting that intracellular, and particularly ER bound CAV1 may be relevant to inter-organelle communication^32^. However, the possible role of the ER in the tumor suppressor activity of CAV1 is completely unknown.

A major function of the ER is the folding and maturation of nascent proteins that transit through the secretory pathway. The loss of ER proteostasis induces the accumulation of unfolded proteins in the lumen of this organelle, a condition referred to as ER stress. Perturbations to ER function in turn activate a series of adaptive mechanisms, collectively known as the unfolded protein response (UPR)^33–35^. If these pro-survival mechanisms are insufficient, a switch in signaling mechanisms occurs to favor pro-apoptotic responses that lead to death of irreversibly damaged cells^36^. Therefore, fine-tuning of adaptive and terminal UPR responses is necessary to sustain cell function, reflected in the contribution of chronic ER stress to diverse diseases^35,37^. In fact, over the past few years, the UPR has become a central actor in cancer development^38^, thus representing an attractive therapeutic target for different types of tumors^39–41^. Extrinsic factors such as hypoxia, nutrient deprivation and acidosis alter the normal function of the ER^42^. Intrinsic stress involved in oncogenic transformation also triggers abnormal levels of ER stress due to the high demand in protein production caused by oncogenic activation, alterations in chromosome number and exacerbated secretory capacity^43^. Moreover, genomic instability, increased mutation rate and redox imbalance further perturb global proteostasis. Overall, accumulating evidence indicates that the UPR influences many hallmarks of cancer^44^, representing an interesting target for cancer treatment.

Three major sensors initiate the UPR, including inositol-requiring enzyme 1-α (IRE1α), protein kinase RNA-like ER kinase (PERK), and activating transcription factor 6 (ATF6)^34^. The activation of both IRE1α and PERK requires their dimerization, oligomerization and *trans*-autophosphorylation^39^. Activated IRE1α excises a 26-nucleotide intron of the mRNA that encodes the transcription factor X-box binding protein 1 (XBP-1), leading to the expression of a more stable and active form known as XBP1s^33^. XBP1s functions as a transcription factor that transactivates several target genes involved in protein folding, ER-associated protein degradation (ERAD), protein translocation to the ER, and protein secretion^40,45^. Activated PERK phosphorylates eukaryotic translation initiator factor 2α (eIF2α), which leads to the inhibition of global protein translation and synthesis, thereby reducing the number of proteins entering the ER, but increasing the cap-independent translation of other mRNAs, such as activating transcription factor 4 (ATF4)^33,35^, which regulates the expression of genes involved in redox control and apoptosis, including the transcription factor C/EBP-homologous protein (CHOP)^46,47^.

Overall, multiple cellular and animal models of cancer, in addition to studies in human tumors have demonstrated the activation of the UPR in the disease^48^ and the role of individual core components of the UPR in tumor growth (reviewed in refs. ^39,42,49^). Most of the evidence available implicates ER stress signaling in the adaptation and survival of cancer cells to the stressful conditions, contributing to oncogenic transformation and the selection of more aggressive cancer cells^44,48^.

In summary, the available evidence posits the UPR, and particularly the IRE1α and PERK signalling branches, as attractive pathways to target for the discovery and development of drugs that may be used to treat cancer^41,50^. Here, we determined whether tumor suppression by CAV1 is linked to modulation of the UPR. Using cell culture and in vivo models of melanoma, we provide evidence indicating that the expression of CAV1 strongly represses the ability of cancer cells to engage the UPR, ablating an adaptive capacity to cope with ER stress. Our results uncover a novel mechanism connecting CAV1 with a central node of the proteostasis network that mediates the growth of solid tumors.

## Materials and methods

### Antibodies and reagents

Polyclonal anti-Caveolin-1 (CAV1; #610060) and monoclonal anti-E-cadherin (#610405) antibodies were from Transduction Laboratories (Lexington, KY, USA). Anti-β-actin antibody was from Sigma-Aldrich (#A5316; St. Louis, MO, USA). Rabbit anti-PERK (#3192), IRE1α (#3294) and phospho-PERK(Thr980) (P-PERK(Thr980)) (#3179) antibodies were from Cell Signaling (Beverly, MA, USA). Goat anti-rabbit immunoglobulin G (IgG) and Goat anti-mouse IgG antibodies coupled to horseradish peroxidase were from Bio-Rad Laboratories (Hercules, CA, USA) and Sigma-Aldrich, respectively. Bicinchoninic acid was from Pierce (Rockford. IL). EZ-ECL chemiluminescent substrate and fetal bovine serum (FBS) were from Biological Industries (Kibbutz BeitHaemek, Israel). TRizol reagent was from Invitrogen Life Technologies (Carlsbad, CA, USA). Hygromycin was from Calbiochem (La Jolla, CA, USA). Cell medium was from Gibco-BRL (Paisley, Scotland, United Kingdom). Anti-BiP (#sc-1050), ATF4 (#sc-390063), CHOP (#sc-7351) and KDEL (#sc-58774) antibodies were from Santa Cruz (Santa Cruz, CA, USA). Anti-Golgin-97 (#A21270), Alexa fluor-546 goat anti-rabbit (#A11010) and Alexa fluor-488 goat anti-mouse (#A11029) antibodies were Thermo Fisher Scientific (Waltham, MA, USA). Tunicamycin (Tm) was purchased from Calbiochem EMB Bioscience (Billerica, MA, USA).

### Cell culture

Metastatic murine melanoma cells B16F10 (ATCC, #CRL6475) expressing or not CAV1^26^, were maintained in RPMI-1640 medium supplemented with 10% FBS and antibiotics (100 U/mL penicillin and 100 mg/mL streptomycin). Cells were cultured at 37 °C and 5% CO_2_. In addition, the metastatic human breast cancer cell line MDA-MB-231 (ATCC, #HTB-26) was stably transfected with short hairpin RNA (shRNA) constructs (pLKO.1-sh-5 (CAV1) or pLKO.1-sh-sc (Scramble; control (Cnt)), as previously described^51^. MDA-MB-231 (shCAV1 and shCnt) cells were maintained in DMEM/F12 medium supplemented with 10% inactivated FBS and antibiotics (100 U/mL penicillin and 100 μg/mL streptomycin). All cells were cultured at 37 °C and 5% CO_2_.

### Site directed mutagenesis of Caveolin-1

In order to clone different CAV1 mutants, pPCR-Script SK(+) (Stratagene, Santa Clara, CA) was used according to manufacturer instructions. In each primer designed to generate the different mutants, an enzyme restriction site was added to facilitate analysis. In the case of CAV1(S80A) and CAV1(S80E), a KpnI site was included, while for CAV1(W98F) and CAV1(W128F) mutants, a NotI site was added.

### B16F10 melanoma cell transfection

Cells transfected with the plasmids pLacIOP (referred to as B16F10(Mock)) and pLacIOP-Caveolin-1 (referred to as B16F10(CAV1)) were previously described^13^. B16F10 cells were grown to 50–60% confluence in a 6 multi-well plate and then stably transfected with 4 μg of pLacIOP-CAV1(S80A) (referred to as CAV1/S80A), pLacIOP-CAV1(S80E) (referred to as CAV1/S80E), pLacIOP-CAV1(W98F), (referred to as CAV1/W98F) and pLacIOP-CAV1(W128F) (referred to as CAV1/W128F); generated as described and transfected using FuGene HD reagent following instructions of the manufacturer. After transfection, cells were plated in complete RPMI medium containing hygromycin (750 μg/mL) for 2 to 3 weeks to yield stably transfected B16F10(CAV1/S80A), B16F10(CAV1/S80E), B16F10(CAV1/W98F), and B16F10(CAV1/W128F) cells, respectively.

### Expression of Caveolin-1 mutant proteins

After cloning different mutants in pPCR-Script SK(+), the construct was excised using NotI enzyme and sub cloned in pLacIOP system, the cloning in the ORF was checked using the following primers: SN: 5’-CCG AGC GCG GCC GCC ATG TCT GGG GGC AAA TAC-3’ and AS: 5’-TTGTCTCCTTCCGTGTTTCA-3’. Once the ORF cloning direction was checked, B16F10 cells were transfected with pLacIOP: B16F10 cell transfected with either, pLacIOP-Caveolin-1 (CAV1/WT) or pLacIOP-Caveolin-1 mutants (CAV1/S80A, CAV1/S80E, CAV1/W98F, CAV1/W128F). Cells were grown in the absence (−) or presence (+) of isopropyl β-D-1-thiogalactopyranoside (IPTG; 1 mM) for 48 h. Then, total protein extracts were obtained and CAV1 was detected by Western blotting and β-actin was included as a loading control to quantify expression levels.

### Animals

All animal experiments were conducted in accordance with ethics protocols approved by the Faculty of Medicine, University of Chile Animal Ethics Committee (CBA 0145 FMUCH and CBA 0417 FMUCH). Species Mus musculus, strain C57-BL/6 (male/female, aged 6–8 weeks at the beginning of the experiments, weighing 20–25 g). The minimum number needed to obtain a statistical power of 95% (1-β) was 4 animals per group, considering a statistical analysis with a significance of α = 0.05. Twelve animals will at least be considered per group, having a total of 48 mice. For in vitro experiments, power calculations were performed based on the work of Lobos-Gonzalez et al. (2013)^26^ and Ortiz et al. (2016)^52^.

### Tumor growth assay

B16F10 cells (which do not express E-cadherin) were stably transfected using FuGene system, with either pLacIOP (Mock), pLacIOP (CAV1) or pLacIOP (CAV1/S80A, CAV1/S80E, CAV1/W98F and CAV1/W128F), and grown in the absence (−) or presence (+) of IPTG (1 mM) for 48 h. Then, cells were collected and suspended in 0.9% NaCl for subcutaneous injection into C57BL/6 mice. Subcutaneous injection into C57BL/6 mice (300000 cells per experiment) was performed by technical personnel blinded to the gene profile of B16F10 inoculated. After subcutaneous injection, tumor formation was monitored up to 15 days when mice were sacrificed. Tumor volume was calculated according to the following formula (*d*)^2 ^× (*D*) × 3,14/6, where-(*d*) represents the lowest radius and (*D*) the highest radius value^26^.

### Same size tumor growth assay

The assay describe above was performed under the similar conditions, except that tumors formed by CAV1 expressing cells were allowed to grow to a similar size (1500–1800 mm^3^) as those formed day 15 by B16F10(Mock) cells. In this case, mice were sacrificed at roughly day 20 and tumors were processed for further analysis.

### Western blotting

Cell extracts were prepared and separated by Sodium Dodecyl Sulfate-Polyacrilamide Gel Electrophoresis (SDS-PAGE) on 12% acrylamide minigels (Bio-Rad), loading 25 μg total protein per lane and transferred to nitrocellulose as previously described^13^. Blots were blocked with 5% milk in phosphate buffered saline (PBS) and probed with anti-β-actin (1:5000), anti-BiP (1:3000), anti-PERK (1:1000), anti IRE1α (1:1000), anti-P-PERK(Thr980) (1:1000), anti-CHOP (1:3000), anti-ATF4 (1:3000), and anti-Caveolin-1 (1:5000) antibodies. Bound antibodies were detected with horseradish peroxidase-conjugated secondary antibodies and using the enhanced EZ-ECL chemiluminescence system.

### Analysis of mRNA levels by qPCR

Total mRNA was isolated using TRIzol reagent following the manufacturer instructions. RNA samples characterized by electrophoresis in 1% agarose gels (quality control) were employed as templates to generate cDNA. cDNAs were amplified by PCR. All reactions involved consecutive 1 min steps at 95 °C (dissociation), 1 min at 55 °C (annealing) and elongation at 72 °C. PCR primers specific for the spliced sequences in *XPB1* mRNA (5’-ACACGCTTGGGAATGGACAC-3’ and 5’-CCATGGGAAGATGTTCTGGG-3’), for *chop* (5’-GTCCCTAGCTTGGCTGACAGA-3’ and 5’-TGGAGAGCGAGGGCTTTG-3’), for *bip* (5’-TCATCGGACGCACTTGGAA-3’ and 5’-CAACCACCTTGAATGGCAAGA-3’) and for the housekeeping gene *β-actin* (5’-TACCACCATGTACCCAGGCA-3’ and 5’-CTCAGGAGGAGCAATGATCTTGAT-3’) were used in amplification reactions.

### XBP-1 splicing assay

Total mRNA was isolated using TRIzol reagent following the manufacturer instructions. RNA samples were characterized by electrophoresis in 1% agarose gels and bands corresponding to spliced (*s*) and unspliced (*u*) *XBP1* mRNA were analyzed by electrophoresis in 2,5% agarose gels. After quantification of bands by scanning densitometry, the results were expressed as percentage of Splicing (*s*/*s* + *u*) × 100.

### Immunoprecipitation

Human Embryonic Kidney 293 cells (HEK cells) were co-transfected with pMSCV- IRE1α-HA and placIOP alone or with different CAV1 constructs (2 μg total), and after 48 h protein extracts were prepared in Lysis buffer (0.5% NP-40, 350 mM NaCL, 150 mM KCl, 50 mM Tris pH 7.6, 50 mM NaF, 1 mM Na_3_VO_4_, 250 mM phenylmethylsulfonyl fluoride (PMSF), and protease inhibitors). Immunoprecipitations (IP) were performed as described^26^. In brief, HA-tagged IRE1α, protein extracts were incubated with anti-HA antibody-agarose complexes (Roche, Basel, Switzerland), for 4 h at 4 °C, and then washed 3 times. Protein complexes were eluted by heating at 95 °C for 5 min in loading buffer.

### Immunofluorescence, image acquisition and analysis

B16F10(Mock), B16F10(CAV1) and B16F10(CAV1/S80A) were plated onto 12-mm coverslips and treated 48 h with IPTG (1 mM) to induce CAV1 expression. MDA-MB-231 wild-type cells were cultured for 48 h. All cells were fixed in 4% paraformaldehyde, permeabilized in 0.1% Triton X-100 and blocked with 2% bovine serum albumin (BSA). Samples were incubated with primary antibodies in blocking solution for 1 h: anti-Caveolin-1 (1:1000), anti-KDEL (1:200) or anti-Golgin-97 (1:200). Then followed an incubation for 1 h with secondary Alexa fluor-546 goat anti-rabbit or Alexa fluor-488 goat anti-mouse antibodies, both diluted 1:500 in blocking solution. Coverslips were mounted on slides using 10% Mowiol-2.5% 1,4-Diazabicyclo [2.2.2] octane. Samples were photographed using an oil-immersion (63x/1.40) objective with a C2 plus spectral confocal microscope (Nikon Eclipse TI, Tokyo, Japan). For analyses, images were deconvoluted, background-subtracted, thresholded and analyzed using the ImageJ software^53^. Manders’ coefficients, as a measure of colocalization, were obtained using the RoB plugin developed by R. Bravo-Sagua (roberto.bq@gmail.com). An average of 10 cells per image were analyzed.

### Proteasomal degradation assay

B16F10(Mock), B16F10(CAV1) and B16F10(CAV1/S80A) were treated 48 h with IPTG (1 mM) to induce CAV1 expression. MDA-MB-231-shCnt and MDA-MB-231-shCAV1 cell lines were cultured for 24 h and then treated with either the proteasome inhibitor MG132 (100 nM) or the autophagy inhibitor chloroquine (CQ) (30 μM) for 12 h. Then, levels of the UPR sensor proteins IRE1α and PERK were determined by Western blotting and quantified. CAV1 protein expression was standardized to β-actin levels as a control.

### Statistical analysis

The sample size was chosen using Altman’s nomogram. A standardized difference of 2.5 was considered. At a power of 90% and a 5% level of significance, the minimum number of repeats in each group needed to show statistical difference was *n* = 3–5. Data were initially compared using D’Agostino and Pearson omnibus normality test. In all cases, groups with similar variance were statistically compared. For paired groups, a two-tailed test was employed. Data analyzed in this manner are specifically indicated in the corresponding figure legend. All groups compared were from three or more independent experiments. A *p* < 0.05 was considered significant.

## Results

### The tumor suppressor activity of Caveolin-1 correlates with the suppression of UPR signalling in vivo

We have previously determined that CAV1 functions as a tumor suppressor in vivo in melanoma cells even in the absence of known mediators of its tumor suppressor activity, such as E-cadherin^26^. Earlier observations also indicated that CAV1 accumulates in the perinuclear region^26,52^, raising the possibility that CAV1 might be present in the ER and affect the signalling of the UPR.

To further evaluate this possibility, we focused on two metastatic cell models lacking E-cadherin, B16F10 murine melanoma cells that lack CAV1 expression and MDA-MB-231 human breast cancer cells with high endogenous levels of CAV1. The effects of CAV1 expression on the UPR were evaluated either by enforcing its expression using an inducible system in B16F10 cells or by knocking down endogenous CAV1 in MDA-MB-231 cells (Supplementary Fig. S1). Importantly, we confirmed that both B16F10 and MDA-MB-231 cells did not express E-cadherin, using the DLD1 cell line as a positive control (Supplementary Fig. S1). In addition, in both cell models CAV1 was present in the ER/Golgi compartments (Supplementary Figs. S2, S3).

**Fig. 1:**
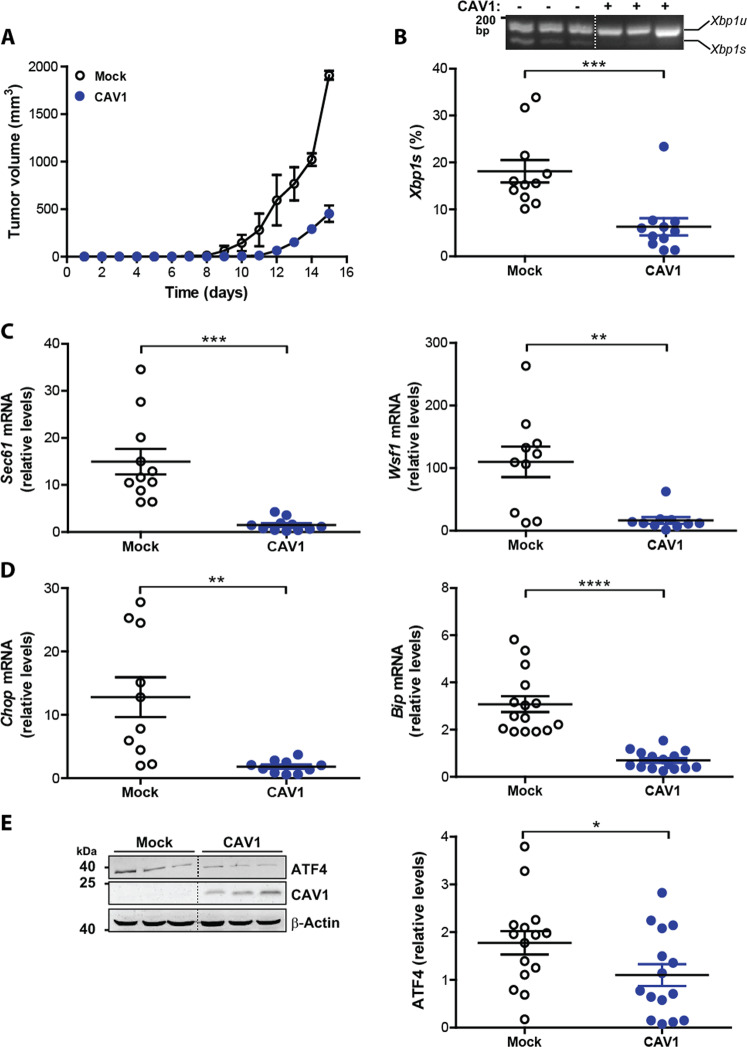
CAV1 mediated inhibition of tumor growth is linked to UPR suppression. B16F10(Mock) or B16F10 (CAV1) cells were cultured for 48 h in the presence of IPTG (1 mM) to induce CAV1 expression. Then 300,000 cells were injected subcutaneously into C57BL/6 mice and tumor development was evaluated up to 15 days when mice were sacrificed. **a** Tumor volumes for each group (*n* = 3, mean ± SEM) are shown from day 1 to 15. **b** Following RNA isolation after tumor growth, the expression of the IRE1α target, *Xbp1* mRNA was evaluated by a RT-PCR based assay and quantified by scanning densitometry. Results are expressed as the percentage of splicing (*s*/*s* + *u*) × 100 (*s*: spliced, *u*: unspliced; *n* = 11, mean ± SEM, Wilcoxon signed rank test, ***p = 0.001). **c** The mRNA levels of additional IRE1α target genes *Sec61* and *Wsf1* were analyzed by qPCR. *Actb* was used as an internal control (*n* = 11, mean ± SEM, paired *t*-test, ***p = 0.0007 and *n* = 10, Wilcoxon signed rank test, ***p = 0.002, respectively). **d** Targets of PERK were also analyzed. *Chop* and *Bip* mRNA were determined by qPCR using *Actb* as an internal control (*n* = 10, mean ± SEM, paired *t*-test, ***p = 0.0076 and *n* = 15, ****p < 0.0001, respectively). **e** ATF4 levels were analyzed by Western blotting after total protein isolation from tumors and quantified by scanning densitometry (*n* = 15, mean ± SEM, paired *t*-test, **p = 0.0136). β-actin was used as loading control.

To test whether CAV1 modulates the UPR during tumor growth, B16F10(Mock) and B16F10(CAV1) melanoma cells were injected subcutaneously into syngenic C57BL/6 mice to evaluate tumor progression in vivo. After 15 days, tumor volumes were on average 1851 mm^3^ and 400 mm^3^ for B16F10(Mock) and B16F10(CAV1) cells, respectively (Fig. 1a). These results confirm previous studies showing that expression of wild-type CAV1 in B16F10 cells delayed tumor formation in C57BL/6 mice^26^.

In order to analyze the activation of IRE1α-dependent signaling, we determined the splicing of *Xbp1* mRNA (Fig. 1b), as well as expression of its target genes including *Sec61* and *Wsf1*^54^ (Fig. 1c). We observed diminished *Xbp1* mRNA splicing in tumors formed by CAV1-expressing cells (Fig. 1b). Also, the mRNA levels of *Sec61* and *Wsf1* were significantly reduced as determined using quantitative PCR (Fig. 1c).

Given our previous data suggesting that CAV1 presence decreased IRE1α-dependent signaling, we determined whether CAV1 also represses the activity of PERK. First, we determined mRNA levels of the ATF4-target genes *Chop and Bip*. Levels of mRNAs of both genes were significantly attenuated in CAV1 tumors compared to control (Fig. 1d). Consistent with these results, ATF4 protein levels showed a trend to be reduced in CAV1-expressing B16F10 cells (Fig. 1e). Taken together, these results show that CAV1 expression in solid tumors reduces downstream UPR signaling.

### Elevated UPR observed in ex-tumor cells is reduced by CAV1 expression

**Fig. 2:**
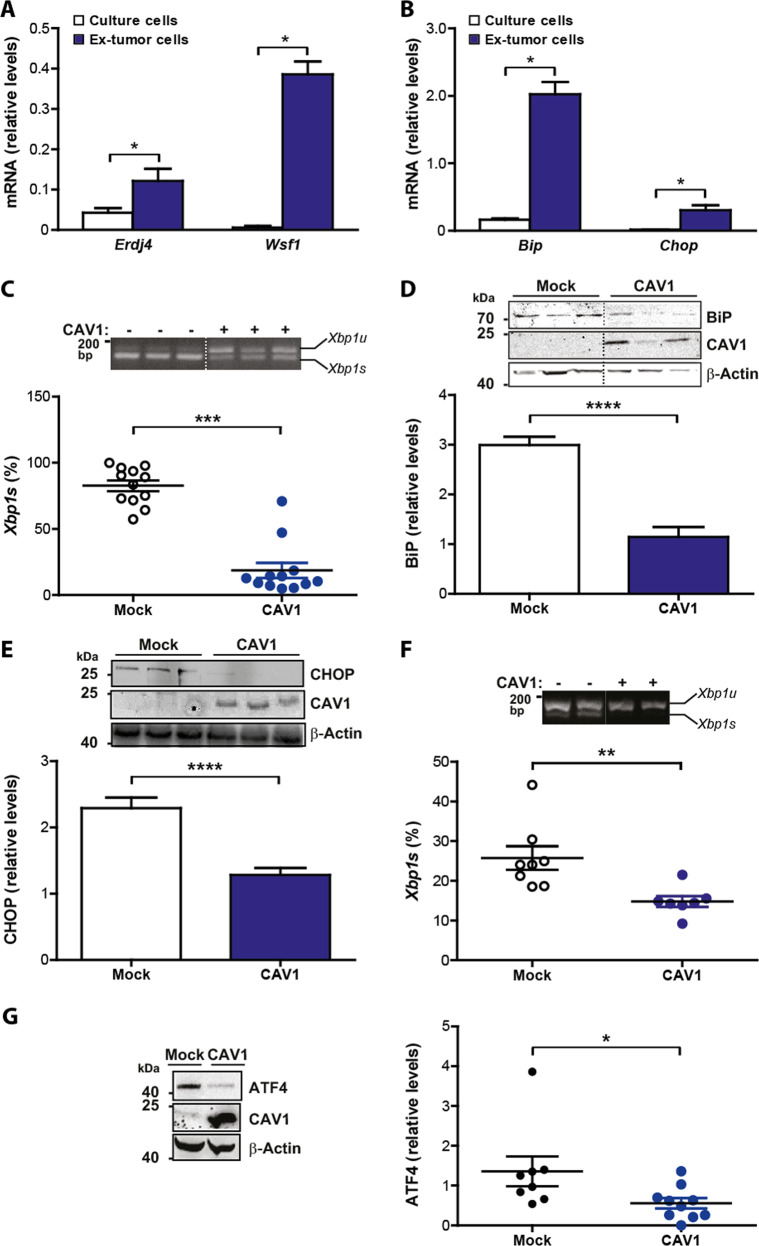
Elevated UPR observed in ex-tumor cells is reduced by CAV1 expression. B16F10(Mock) cells were cultured for 48 h in the presence of IPTG (1 mM). Then 300,000 cells were injected subcutaneously in C57BL/6 mice and tumor development was evaluated up to 15 days when mice were sacrificed. Tumor tissue was trypsinized and isolated cells were cultured for two passages (ex-tumor cells). **a** The expression of IRE1α (*Erdj4* and *Wsf1*) target genes were evaluated by qPCR following RNA isolation before (cells in culture) or after tumor growth (ex-tumor cells). *Actb* was used as internal control (*n* = 6, mean + SEM, Wilcoxon signed rank test, *p = 0.0313). **b** PERK target genes (*Bip* and *Chop*) were evaluated by qPCR following RNA isolation before (cells in culture) or after tumor growth (ex-tumor cells). *Actb* was used as internal control (*n* = 6, mean + SEM, Wilcoxon signed rank test, *p = 0.0313). **c** *Xbp1* mRNA splicing was assessed and quantified as described (see Fig. 1) for cultured and ex-tumor cells (*n* = 12, mean ± SEM, Wilcoxon signed rank test, ***p = 0.0005). **d** Total protein extracts were prepared from cultured and ex-tumor cells and analyzed by Western blotting. The expression and quantification of BiP protein levels are shown (*n* = 20 Mock, *n* = 17 CAV1, mean + SEM, Mann–Whitney test, ****p < 0.0001). β-actin was used as loading control. **e** Total protein extracts were prepared from cultured and ex-tumor cells and analyzed by Western blotting. The expression and quantification of CHOP protein levels are shown (*n* = 9, mean + SEM, unpaired *t*-test, ****p < 0.0001). β-actin was used as loading control. **f** Tumors were grown to the same size/volume (up to ~20 days). *Xbp1* mRNA splicing was assessed and quantified (*n* = 8 Mock, *n* = 7 CAV1, mean ± SEM, Mann–Whitney test, **p = 0.0022). **g** Tumors were grown to the same size/volume (up to ~20 days). PERK-downstream signaling was assessed by determining ATF4 levels by Western blotting after total protein isolation from tumors and quantified by scanning densitometry (*n* = 8 Mock, *n* = 10 CAV1, mean ± SEM, Mann–Whitney test, *p = 0.0205). β-actin was used as loading control.

To ensure that the data obtained using tumor-derived tissue reflected a response elicited predominantly in tumor cells rather than the stromal compartment, we next asked whether suppression of the UPR by CAV1 expression was maintained in melanoma cells isolated from tumors (ex-tumor). To that end, tumors were disaggregated to culture melanoma cells up to 2 passages and then characterized. Using this setting, we observed that CAV1 expression was still inducible with IPTG in these B16F10(CAV1) cells (Supplementary Fig. S4). Importantly, RNA levels of *Erdj4* and *Wsf1* were significantly elevated in ex-tumor cells (Fig. 2a), as compared to cultured cells before tumor formation. Similar results were obtained when *Bip* and *Chop* mRNA levels were analyzed (Fig. 2b). Then, we asked whether CAV1 expression in ex-tumor cells affected IRE1α dependent signaling. We observed reduced *Xbp1* mRNA splicing in B16F10(CAV1) compared to B16F10(Mock) ex-tumor cells (Fig. 2c). Also, protein expression of BiP (Fig. 2d) and CHOP (Fig. 2e) were significantly reduced. These results indicate that the UPR remains elevated in essentially pure ex-tumor melanoma cell populations obtained after 2 passages and that, under these conditions, CAV1 still subdues signaling downstream of IRE1α and PERK.

Tumor growth takes place in hypoxic conditions, where cancer cells adapt to the adverse environment in part by activating the UPR^44^. A caveat to the previous experiments was that the tumors analyzed were collected at the same time point and had different sizes. Thus, the effects of CAV1 on UPR may be the natural consequence of different tumors sizes and volumes at day 15. To rule out this possibility, tumors were grown to the same size/volume, which necessarily implicated extending growth periods to roughly 20 days for tumors formed by B16F10(CAV1) cells. The average size for both groups of tumors was 1500 mm^3^. The results obtained revealed that under these circumstances, both UPR branches mediated by IRE1α and PERK were significantly attenuated by the expression of CAV1 (Fig. 2f, g). In all cases, CAV1 expression coincided with reduced levels of UPR markers. Thus, these results demonstrate that reduced UPR signaling observed in CAV1-expressing cells in tumors was independent of the tumor volume.

### Attenuated UPR signaling in cells undergoing ER stress or hypoxia upon CAV1 expression

**Fig. 3:**
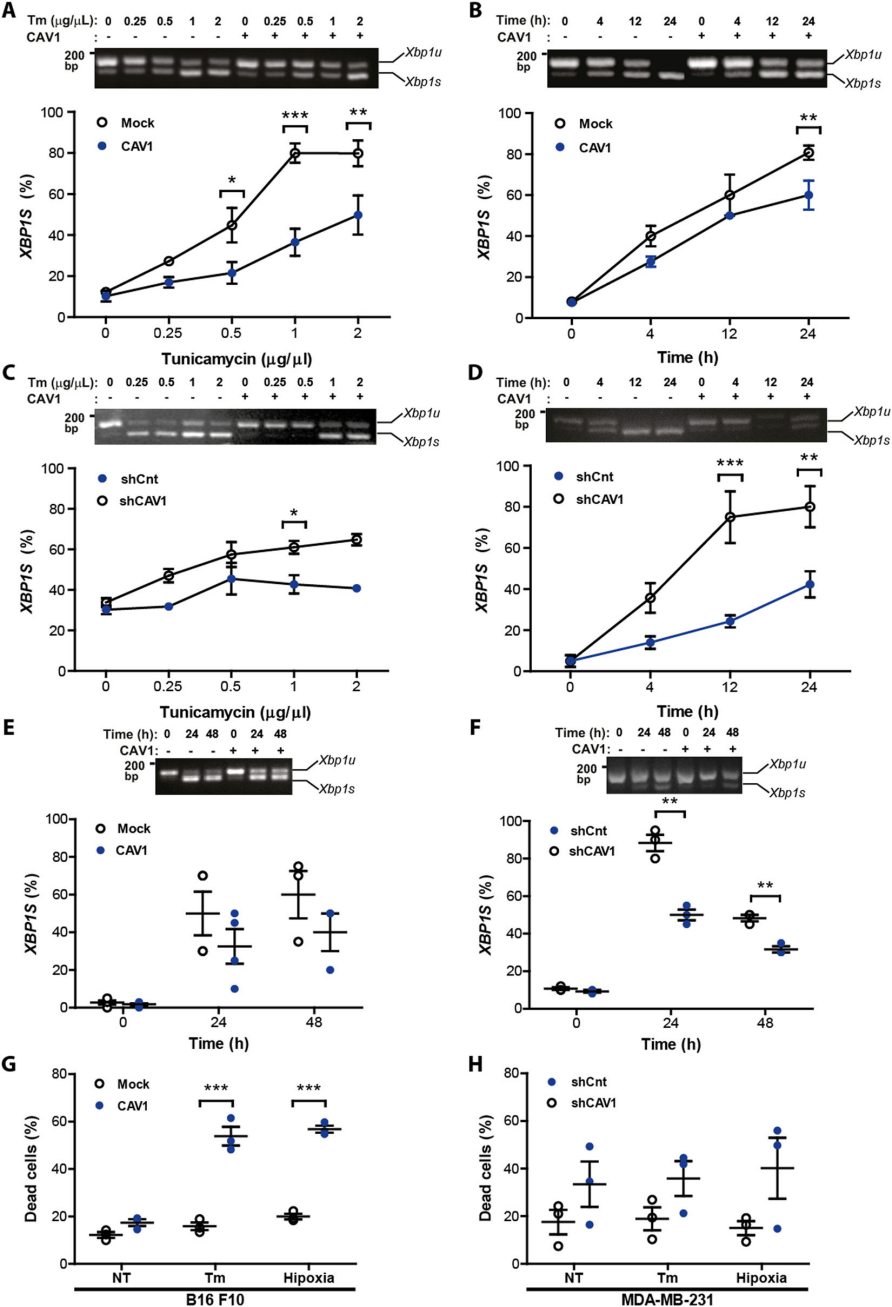
CAV1 expression reduced tunicamycin and hypoxia induced UPR and increased cell death in B16F10 and MDA-MB-231 cells. B16F10(Mock) and B16F10(CAV1) cells were cultured in RPMI supplemented with FBS 10% for 48 h in the presence of IPTG (1 mM). MDA-MB-231 shCnt or shCAV1 cells were cultured in DMEM/F12 medium supplemented with 10% FBS for 48 h. Afterwards, both cell lines were treated with Tm or exposed to hypoxia. **a** B16F10(Mock) and B16F10(CAV1) cells were treated with increasing concentrations of Tm (0.25–2 μg/μL) for 4 h. Then, cells were collected, and total RNA was obtained. Signaling downstream of IRE1α was monitored by quantifying *XBP1* mRNA splicing as described (*n* = 3, mean ± SEM, two-way repeated measures ANOVA, *p < 0.05, **p < 0.01, ***p < 0.001; mean = line). **b** Cell lines shown in **a** were treated with 0.5 μg/μL Tm up to 24 h and *XBP1* mRNA splicing was quantified as described (*n* = 4, mean ± SEM, two-way ANOVA, **p < 0.01). **c** MDA-MB-231(shCnt) or MDA-MB-231(shCAV1) cells were also evaluated as indicated above. Representative results in MDA-MB-231 cells for *XBP1* splicing using increasing concentrations of Tm (*n* = 3, mean ± SEM, two-way ANOVA, *p < 0.05) **d** MDA-MB-231(shCnt) or MDA-MB-231(shCAV1) cells were treated with 0.5 μg/μL Tm up to 24 h and *XBP1* mRNA splicing was quantified as described (*n* = 3, mean ± SEM, two-way repeated measures ANOVA, **p < 0.01, ***p < 0.001). **e** B16F10(Mock) and B16F10(CAV1) cells were cultured for 48 h and then exposed to hypoxia (1% O_2_) for 24 or 48 h. IRE1α signaling was assessed by the *XBP1* splicing assay (*n* = 4, mean ± SEM). **f** The same experiments described in **e** were carried out in the MDA-MB-231 cell line (*n* = 3, mean ± SEM, two-way repeated measures ANOVA, **p < 0.01, ***p < 0.001). **g** B16F10(Mock) and B16F10(CAV1) cells were treated with Tm (0.5 μg/μL) or exposed to hypoxia for 24 h and cell death was determined by flow cytometry analysis following propidium iodide staining (*n* = 3, mean ± SEM, two-way repeated measures ANOVA, ***p < 0.001, mean ± SEM). **h** MDA-MB-231(shCnt) and MDA-MB-231(shCAV1) cells were treated with Tm (0.5 μg/μL) or exposed to hypoxia for 24 h and cell death was determined by flow cytometry analysis following propidium iodide staining (*n* = 3, mean ± SEM).

To study the possible direct involvement of CAV1 in the control of the UPR, we developed cell culture studies to induce experimental ER stress. To that end, melanoma cell lines were treated either with the ER stress agent tunicamycin (Tm, inhibitor on N-linked glycosylation) or exposed to hypoxia (Fig. 3). Using dose-response experiments, we observed that the expression of CAV1 resulted in reduced ER stress signals upon Tm treatment in both B16F10 and MDA-MB-231 cells (Fig. 3a, c). Likewise, in B16F10 cells treated with 0.5 μg/μL Tm for up to 24 h, CAV1 expression resulted in reduced *XBP1* mRNA splicing compared to B16F10(Mock) cells (Fig. 3b). In addition, knocking down CAV1 in MDA-MB-231 cells exacerbated the activation of the UPR in cells treated with Tm in dose-response (Fig. 3c) and time course experiments (Fig. 3d).

Then, we exposed melanoma cells to hypoxic conditions up to 24 h. The results obtained in B16F10 cells virtually recapitulated the observations obtained with tunicamycin treatment. The expression of CAV1 translated into lower levels of *XBP1* mRNA splicing after 48 h (Fig. 3e), whereas in MDA-MB-231 cells knocking down CAV1 increased *XBP1* mRNA splicing significantly (Fig. 3f). Finally, to determine the functional consequences of the modulatory effects of CAV1 on the UPR, we monitored the ability of cells to cope with ER stress by determining the survival of cells under stress. To that end, we quantified apoptotic cell death using FACS analysis in both cell models after inducing ER stress. Remarkably, elevated levels of CAV1 expression resulted in enhanced death in both B16F10 and MDA-MB-231 cells (Fig. 3g, h, respectively) in response to either hypoxia or Tm treatment for 24 h. Thus, the capacity of CAV1 to suppress the activation of the UPR under stress correlates with a higher susceptibility to undergo cell death.

### Serine-80 of Caveolin-1 mediates the repression of the UPR

**Fig. 4:**
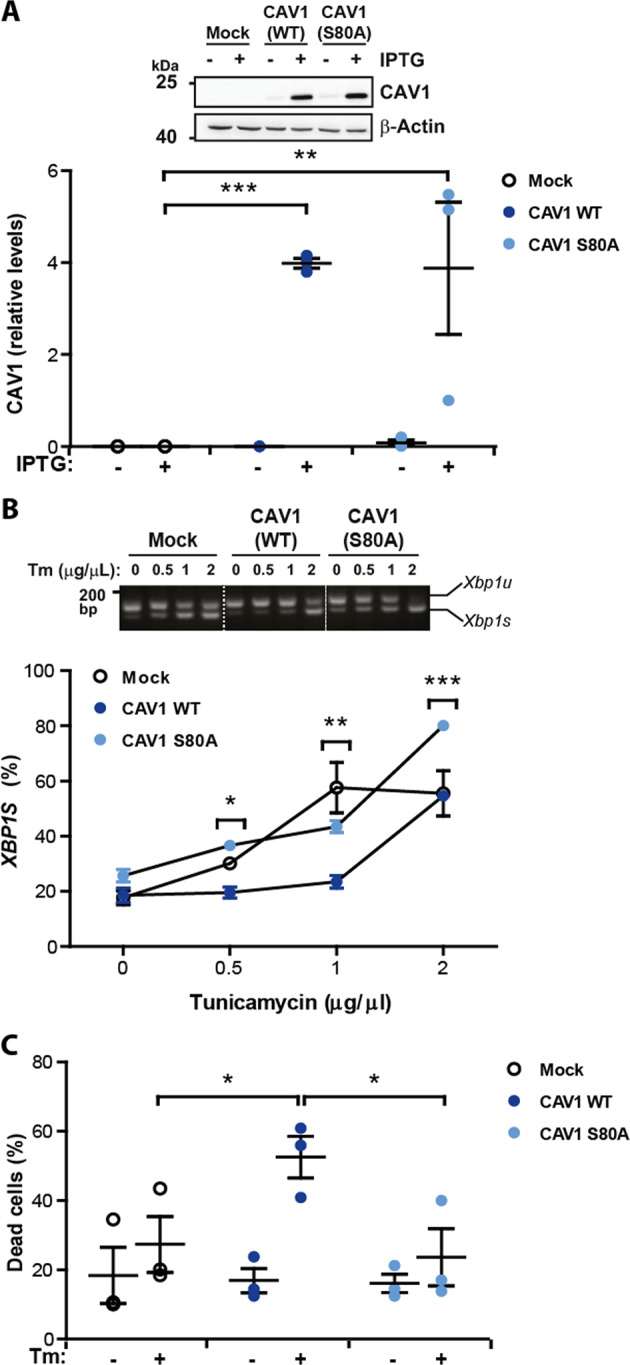
UPR suppression and increased cell death mediated by CAV1 is prevented by the S80A mutation. B16F10(Mock), B16F10(CAV1) or B16F10 cells expressing CAV1 mutant S80A (B16F10(CAV1/S80A)) were grown in the presence of IPTG (1 mM) for 48 h. **a** Total protein extracts were obtained and levels of CAV1 were evaluated by Western blotting and quantified. β-actin was included as a loading control (*n* = 3, mean ± SEM, two-way repeated measures ANOVA, **p < 0.01, ***p < 0.001). **b** B16F10(Mock), B16F10(CAV1) and B16F10(CAV1/S80A) were then exposed to increasing concentrations of Tm for 4 h (0.25–2 μg/μL). *XBP1* mRNA splicing was detected and quantified as described (*n* = 3, mean ± SEM, two-way repeated measures ANOVA, *p < 0.05, **p < 0.01, ***p < 0.001 between B16F10(CAV1) and B16F10(CAV1/S80A)). **c** The same cell lines described above were exposed to Tm (0.5 μg/μL) for 24 h and then cell death was evaluated following propidium Iodide staining (*n* = 3, mean ± SEM, two-way repeated measures ANOVA, *p < 0.05).

The tumor suppressor activity of CAV1 has been associated with its scaffolding domain (CSD), which is implicated in binding to and inhibiting the activity of numerous signaling proteins. Thus, we performed mutagenesis analysis of the region between the amino acids 80–132 to identify elements of CAV1 that are required to block the UPR. Initially, we were particularly interested in evaluating the role of S80 (Fig. 4a), a highly conserved amino acid residue implicated in the control of CAV1 secretion and its location to the ER^30^. We determined the effects of overexpressing CAV1 wild-type or the S80A mutant in the activation of the UPR. Mutation of S80A in CAV1 ablated its ability to repress the splicing of the *XBP1* mRNA under ER stress (Fig. 4b). Consistent with this result, the expression of CAV1(S80A) in B16F10 cells failed to enhance the susceptibility of B16F10 cells to ER stress-mediated cell death (Fig. 4c) when compared to the wild-type protein.

**Fig. 5:**
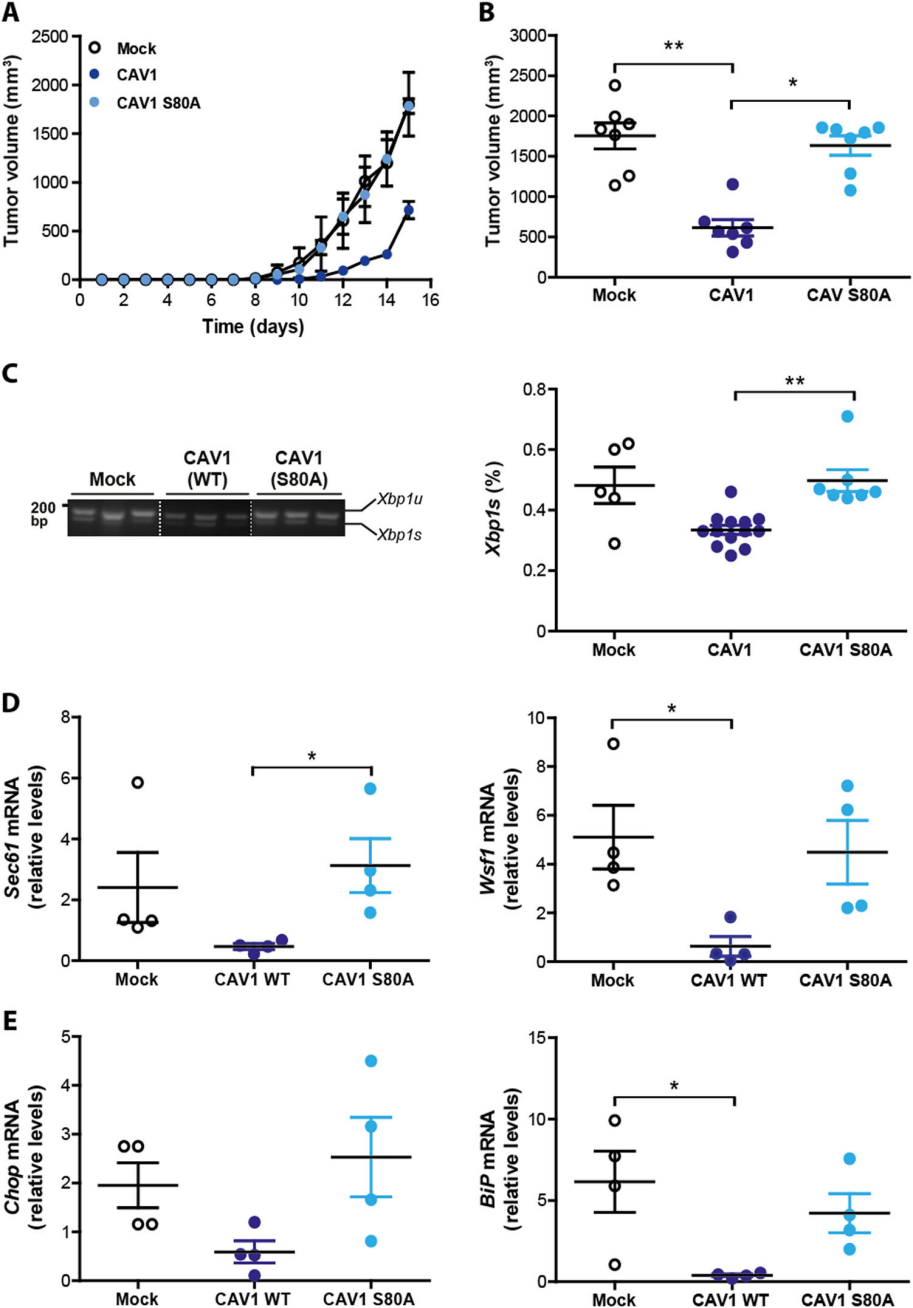
CAV1 mediated inhibition of UPR in tumors requires serine-80. B16F10(Mock), B16F10(CAV1) or B16F10(CAV1/S80A) cells were grown in the presence of IPTG (1 mM) for 48 h to induce CAV1 expression. Then, cells (300,000) were injected subcutaneously into C57BL/6 mice and tumor progression was evaluated up to 15 days when mice were sacrificed. **a** Tumor volumes for each group (*n* = 3, mean ± SEM) were monitored every three days and are shown from day 1 to 15. **b** Tumor volume measured for each group obtained at day 15 (*n* = 7, mean ± SEM, Kruskal–Wallis test, **p = 0.0018, *p < 0.05). **c** At day 15, tumors from each group were processed, total RNA was extracted and *Xbp1* mRNA splicing was detected and quantified by scanning densitometry. Results are expressed as the percentage of splicing (*s*/*s* + *u*) × 100 (*s*: spliced, *u*: unspliced; *n* = 5 Mock, *n* = 13 CAV1, *n* = 7 CAV1/S80A, mean ± SEM, Kruskal–Wallis test, **p = 0.0023). **d** The mRNA levels of the IRE1α target genes *Sec61* and *Wsf1* were evaluated by qPCR. All values were standardized to β-actin used as an internal control (*n* = 4, mean ± SEM, Kruskal–Wallis test, *p < 0.05). **e** Two target genes of the PERK pathway, *Chop* and *Bip* were evaluated by qPCR. All values were standardized to β-actin used as an internal control (*n* = 4, mean ± SEM, Kruskal–Wallis test, *p < 0.05).

We then tested the consequences of mutating S80 in CAV1 in its tumor suppressor activity and its relation to ER stress signaling. Subcutaneous tumor progression was evaluated using B16F10 cells expressing either wild-type CAV1 or CAV1(S80A). As previously described, expression of wild-type CAV1 in B16F10 cells delayed tumor formation in C57BL/6 mice (Fig. 5a). In addition, tumors formed by B16F10 cells expressing wild-type CAV1 were significantly smaller on day-15 post-subcutaneous injection than the tumors from animals injected with B16F10(Mock) cells (Fig. 5a). Remarkably, the introduction of the single S80A mutation fully abrogated the capacity of CAV1 to suppress tumor formation in vivo in B16F10 cells (Fig. 5a). The tumor volume measured for each group obtained at day 15 is shown in Fig. 5b. Importantly, expression levels of CAV1(S80A) were equivalent to the wild-type form (Fig. 4a).

Of note, the analysis of other CAV1 mutations (W98F, W128F) revealed that aberrant behavior was unique to CAV1(S80A) because for the former two cases the tumor suppressor function was maintained (Supplementary Fig. S5). In addition, we observed that CAV1(S80A) distribution was intracellular and diffuse, and that ER stress increased the localization of the S80A mutant in the ER/Golgi compartment of B16F10(CAV1/S80A) cells (Supplementary Fig. S3). Thus, the inability of CAV1(S80A) to suppress the UPR in B16F10 cells does not appear to be attributable to absence from the ER compartment.

To determine the effects of this mutation on UPR signaling in vivo, we evaluated in the same tumor samples whether presence of the CAV1(WT) and CAV1(S80A) proteins correlated with the expected alterations in the UPR. Consistent with our cell culture experiments, the levels of *Xbp1* mRNA splicing were not affected by the S80A mutation, showing similar levels to the ones observed in B16F10(Mock) cells (Fig. 5c). Virtually identical results were obtained when we analyzed the transcription of downstream UPR responses (Fig. 5d, e). Taken together, these findings suggest that mutation of the S80 residue affected the ability of CAV1 to suppress tumor growth of B16F10 melanoma cells, associated with a loss in the capacity of CAV1 to repress UPR signaling.

### CAV1 expression reduces UPR stress sensor activation and forms a protein complex with IRE1α

**Fig. 6:**
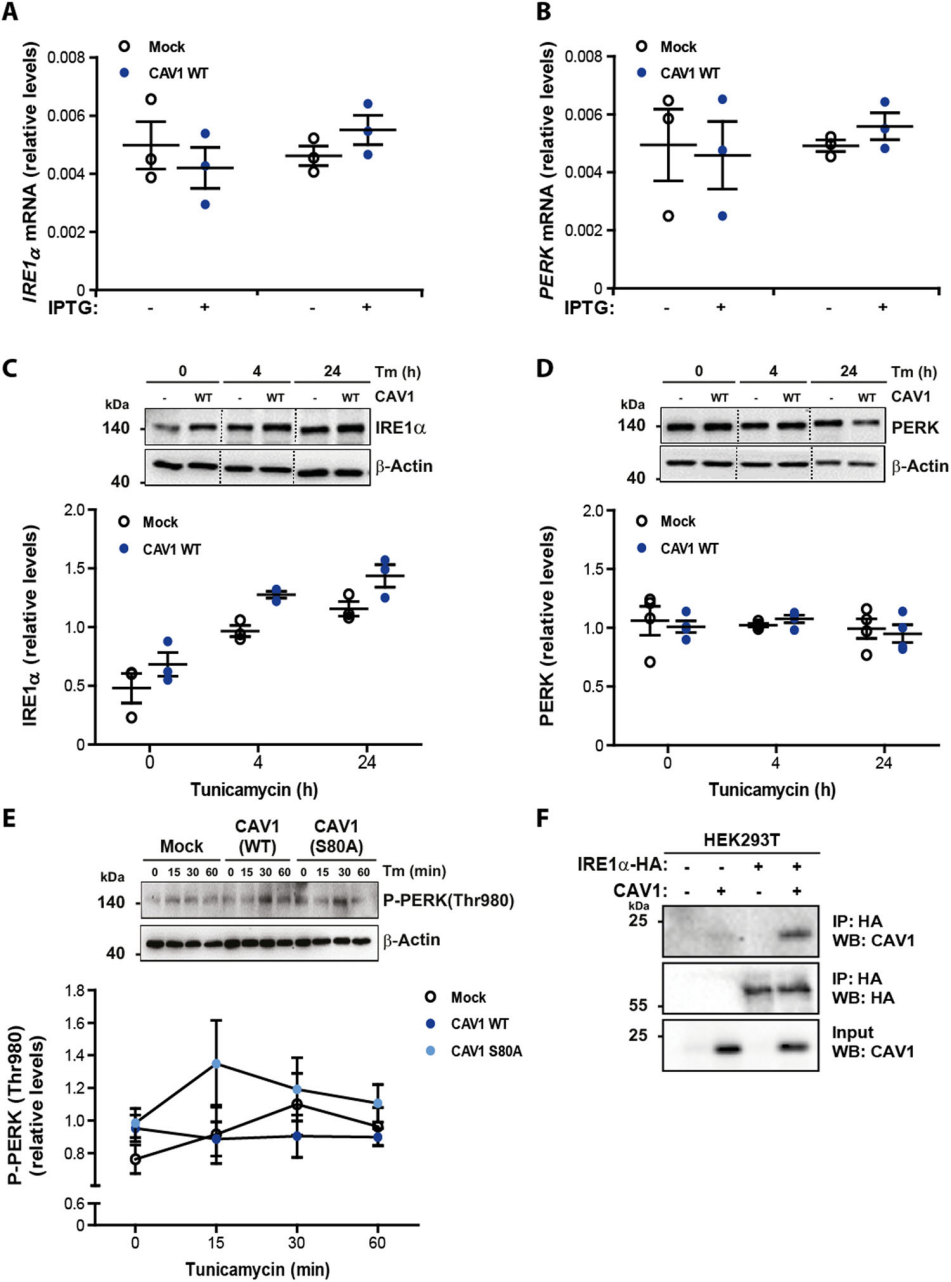
CAV1 expression reduces UPR stress sensor activation and forms a protein complex with IRE1α. B16F10(Mock) or B16F10(CAV1) cells were grown in the presence of IPTG (1 mM) for 48 h to induce CAV1 expression. **a** Total RNA was obtained to evaluate the mRNA levels of the UPR sensor IRE1α. Values were normalized to the housekeeping gene β-actin (*n* = 3, mean ± SEM). **b** Total RNA was obtained to evaluate the mRNA levels of the UPR sensor PERK. Values were normalized to the housekeeping gene β-actin (*n* = 3, mean ± SEM). **c** B16F10(Mock) or B16F10(CAV1) cells were exposed to Tm (0.5 μg/μL) for 4 and 24 h. Total protein extracts were obtained and levels of IRE1α were evaluated by Western blotting and quantified (*n* = 3, mean ± SEM). β-actin was included as a loading control. **d** B16F10(Mock) or B16F10(CAV1) cells were exposed to Tm (0.5 μg/μL) for 4 and 24 h. Total protein extracts were obtained and levels of PERK were evaluated by Western blotting and quantified (*n* = 4, mean ± SEM). β-actin was included as a loading control. **e** B16F10(Mock), B16F10(CAV1) and B16F10(CAV1/S80A) cells were grown in the presence of IPTG (1 mM) for 48 h and then exposed to Tm (0.5 μg/μL) for 0, 15, 30, and 60 min. Total protein extracts were obtained and levels of P-PERK(Thr 980) were evaluated by Western blotting and quantified (*n* = 3, mean ± SEM). β-actin was included as a loading control. **f** HEK293T cells were co-transfected with pMSCV-IRE1α-HA and Mock and CAV1 DNA constructs. IP HA-tagged IRE1α protein extracts were incubated with anti-HA antibody-agarose complexes to detect CAV1 and IRE1α protein complexes. Results shown are representative of *n* = 3 independent experiments.

CAV1 is widely viewed as a negative regulator of signaling, based on observations linking interaction with CAV1 to subsequent inhibition of target protein function. This inhibition can occur via transcriptional or post-transcriptional mechanisms. With this in mind, we were interested in establishing whether CAV1 mediated suppression of the UPR occurred via a transcriptional mechanism. To that end, mRNA levels for the UPR sensors IRE1α and PERK were determined by quantitative PCR in B16F10 cells following expression of CAV1. No significant differences in mRNA encoding for IRE1α and PERK were found upon CAV1 expression (Fig. 6a, b). We then asked whether alternative modes of action could be mediating UPR inhibition by CAV1 and evaluated the protein levels of the IRE1α and PERK sensors. Likewise, induction of ER stress with Tm did not alter total protein levels of IRE1α and PERK in B16F10 cells upon CAV1 expression (Fig. 6c, d, respectively).

We subsequently evaluated whether the functional differences observed may be attributable to degradation events. First, we induced CAV1 expression for 48 h and then cells were treated with either the proteasome inhibitor MG132 (12 h) or the autophagy inhibitor chloroquine (CQ) for 5 h. As depicted in Supplementary Fig. S6A and S6B, no differences in protein levels were observed under these conditions, suggesting that the inhibition of UPR mediated by CAV1 does not occur by enhanced degradation via these two pathways.

We then assessed whether CAV1 expression alters PERK activation kinetics. To this end, we performed a time-course, evaluating the ability of Tm to induce short-term ER stress and analyzed by Western blotting phosphorylated PERK (Thr980) levels as a marker of PERK kinase activation. Our results suggest that the expression of CAV1(WT) in B16 F10 cells delayed PERK kinase activation, compared to B16F10(Mock) cells (Fig. 6e). Importantly, CAV1(S80A) mutation not only restored but even increased PERK activation (Fig. 6e). Therefore, it is likely that CAV1 exerts its tumor suppressor activity by altering the activation kinetics of UPR sensors like PERK in the ER.

Furthermore, we evaluated the possible protein complex formation between CAV1 and IREα. To assess this, HEK293T cells were co-transfected with expression vectors for HA-tagged IRE1α and CAV1 or empty vector. IRE1α co-immunoprecipitated with CAV1(WT) (Fig. 6f). Taken together these results suggest that one potential mechanism, by which CAV1 expression exerts its tumor suppressor activity, is by forming protein complexes with UPR sensors in the ER.

## Discussion

The UPR signaling network is emerging as an interesting driver of several cellular processes involved in cancer development. Most evidence available suggests that the UPR operates as a pro-oncogenic mechanism that increases cell adaptation and survival to cope with both major intrinsic changes driven by oncogenes and adverse environments to which tumor cells are subjected^55^. Hypoxia, nutrient deprivation, acidosis and the high demand of protein production generate a global ER stress state that engages all three UPR branches^43^. Here we report for the first time, that the tumor suppressor activity of CAV1 is linked to the repression of the ability of cells to engage adaptive programs to cope with ER stress. Moreover, UPR inhibition by CAV1 was abrogated by site-directed mutagenesis of serine-80, an amino acid implicated in CAV1 secretion and its location to the ER.

During tumor formation, the UPR is induced possibly as an essential adaptive response of cancer cells^56,57^. Importantly, depending on the intensity of the stress stimuli the UPR will favor survival responses or, alternatively, elimination of damaged cells by apoptosis^58,59^. Here we report that the tumor suppressor activity of CAV1 is associated with a modulation of the activation of the UPR as shown in cell culture paradigms of ER stress and melanoma models in vivo. The amplitude and kinetics of ER stress signaling are regulated by the binding of different co-factors to the cytosolic region, post-translational modifications, and proteasomal degradation^36^. IRE1α and PERK can be viewed as scaffolds onto which many components assemble to modulate their activity and instigate specific signaling outputs, a platform referred to as the *UPRosome*^60^. Under prolonged ER stress, IRE1α is turned off involving the physical interaction with several factors previously linked to the regulation of apoptosis as we previously reported^54,61^. We speculate that CAV1 may reduce the activity of the UPR through direct binding and repression of the activity of IRE1α.

Our current work, particularly in B16F10 cells, which do not express Cavin1 and hence lack morphologically distinguishable caveolae^52^, further reinforces the notion that CAV1, beyond its structural/functional role at the plasma membrane, is important in the control of intracellular functions, particularly in the ER^62,63^, where CAV1 participates in processes that regulate cell death^64^. Indeed, CAV1 is located at the mitochondria-associated ER membrane fraction (MAMs)^31^ and genetic ablation of CAV1 has detrimental effects on mitochondrial function^64,65^. Moreover, we recently showed that CAV1 impairs the remodeling of ER-mitochondria contacts by precluding protein kinase A signaling, as evidenced by alterations in organelle distribution and impaired phosphorylation of the GTPase dynamin-related protein 1, thus enhancing cell death in response to ER stress^66^. Alternatively, CAV1 has recently been implicated as a core component of the calcium-dependent apoptotic pathway that regulates critical mitochondrial functions during tumor development^67^. These observations raise the possibility that tumor suppression by CAV1 is due to inhibition of ER-mitochondria communication, and implicate the UPR receptors PERK and IRE1α in these events.

The fact that S80 mutation ablated the inhibitory effects of CAV1 on the UPR suggests that CAV1 exerts these effects in a location-specific manner at the ER, and that accumulation of CAV1 in the ER modulates the UPR. CAV1 reportedly functions as a scaffolding protein that binds to and inhibits a large number of proteins via CSD-CBD interactions^10,20^. Lisanti et al. (2005) proposed that CAV1 post-translational modifications at S80 could induce topological changes, thereby converting CAV1 into a protein that is secreted through the classical secretory pathway^20^. Moreover, S80A mutation led to loss of the ability of CAV1 to inhibit the UPR, suggesting that S80 is important for the ability of CAV1 to suppress tumor growth.

CAV1 is also present in the ER and specifically enriched at MAMs, and its ablation greatly reduces ER-mitochondria contact sites while increasing inter-organelle cholesterol transfer^31^. Importantly, the stability of MAMs directly influences the kinetics and amplitude of UPR responses, correlating with the presence of PERK and IRE1α in these membrane subdomains where they regulate bioenergetics^68,69^. These observations agree with previous studies showing CAV1 presence in lipid raft-like domains of the ER^70^, and its protective role against mitochondrial dysfunction induced by cholesterol overload^64^. However, whether CAV1 modulates PERK activation required for the regulation of ER-mitochondria communication remains unexplored. Another possibility that may link CAV1 with the UPR is the modulation of cholesterol biosynthesis. CAV1 is well known to control transport and secretion of cholesterol^71^, whereas intracellular cholesterol accumulation triggers ER stress^71^. Moreover, the activity of the UPR has been also linked to cholesterol deposition in various models^72^. While extremely interesting, these possibilities need to be addressed in future work beyond the scope of this study.

In summary, we conclude that tumor suppression by CAV1 in the cytosol is attributable at least in part to attenuation of the UPR, and identified S80 as an essential residue in this context. The UPR is an adaptive mechanism sustaining the function of specialized secretory cells, but also a process altered in a variety of diseases including diabetes, obesity, autoimmune diseases, degenerative disorders in addition to cancer^35^^,^^37^. Particularly, the involvement of ER stress in cancer highlights the importance of the UPR as a potential therapeutic target.

## Supplementary information

Supplementary Figure

Supplementary Figure

Supplementary Figure

Supplementary Figure

Supplementary Figure

Supplementary Figure

Supplementary Figure legends
