# Supplementary figures and images for: Caveolin-1 suppresses tumor formation through the inhibition of the unfolded protein response
Source: Cell Death Dis. 2020 Aug 3;11(8):648. doi: 10.1038/s41419-020-02792-4 (PMC7434918; doi:10.1038/s41419-020-02792-4)

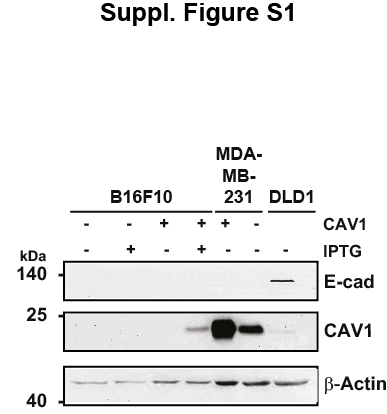

Supplement: Supplementary file 1 — Supplementary Figure [file 41419_2020_2792_MOESM1_ESM.png]

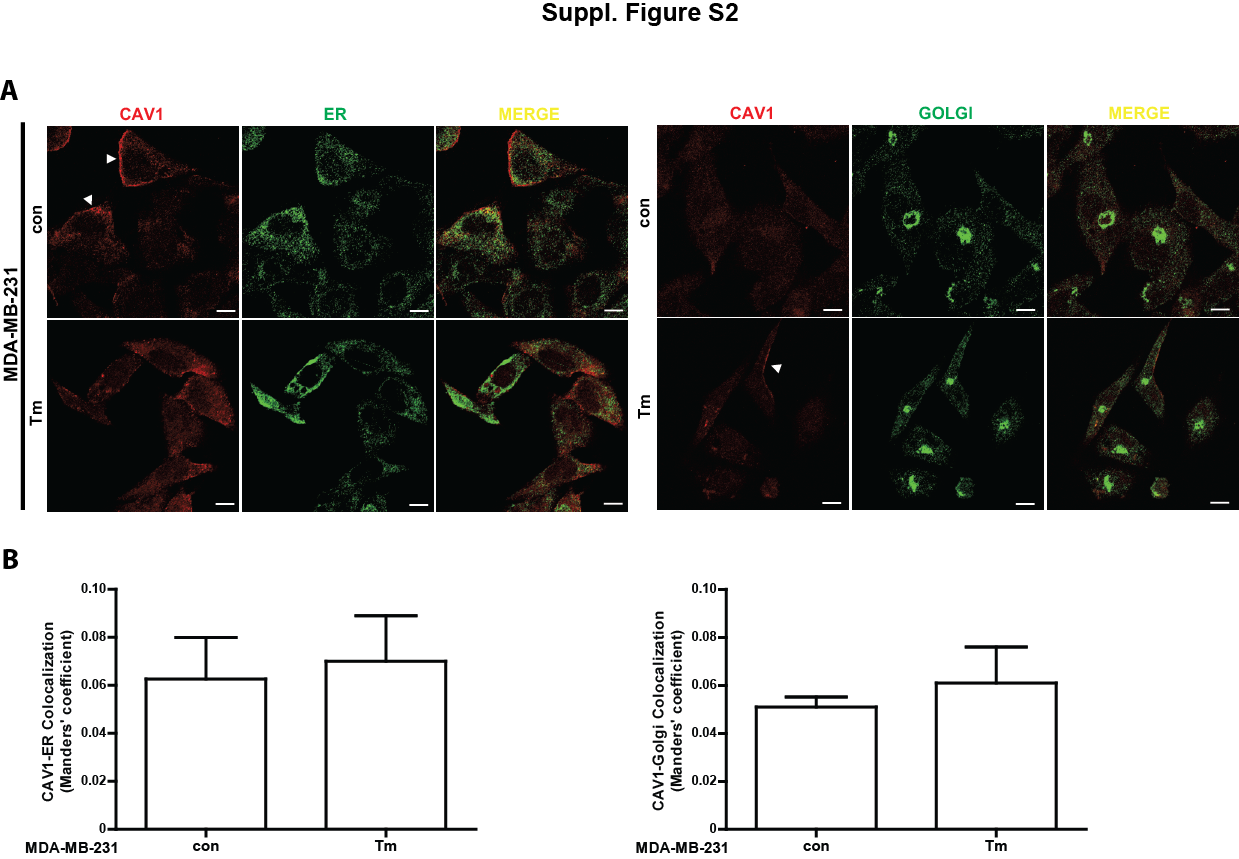

Supplement: Supplementary file 2 — Supplementary Figure [file 41419_2020_2792_MOESM2_ESM.png]

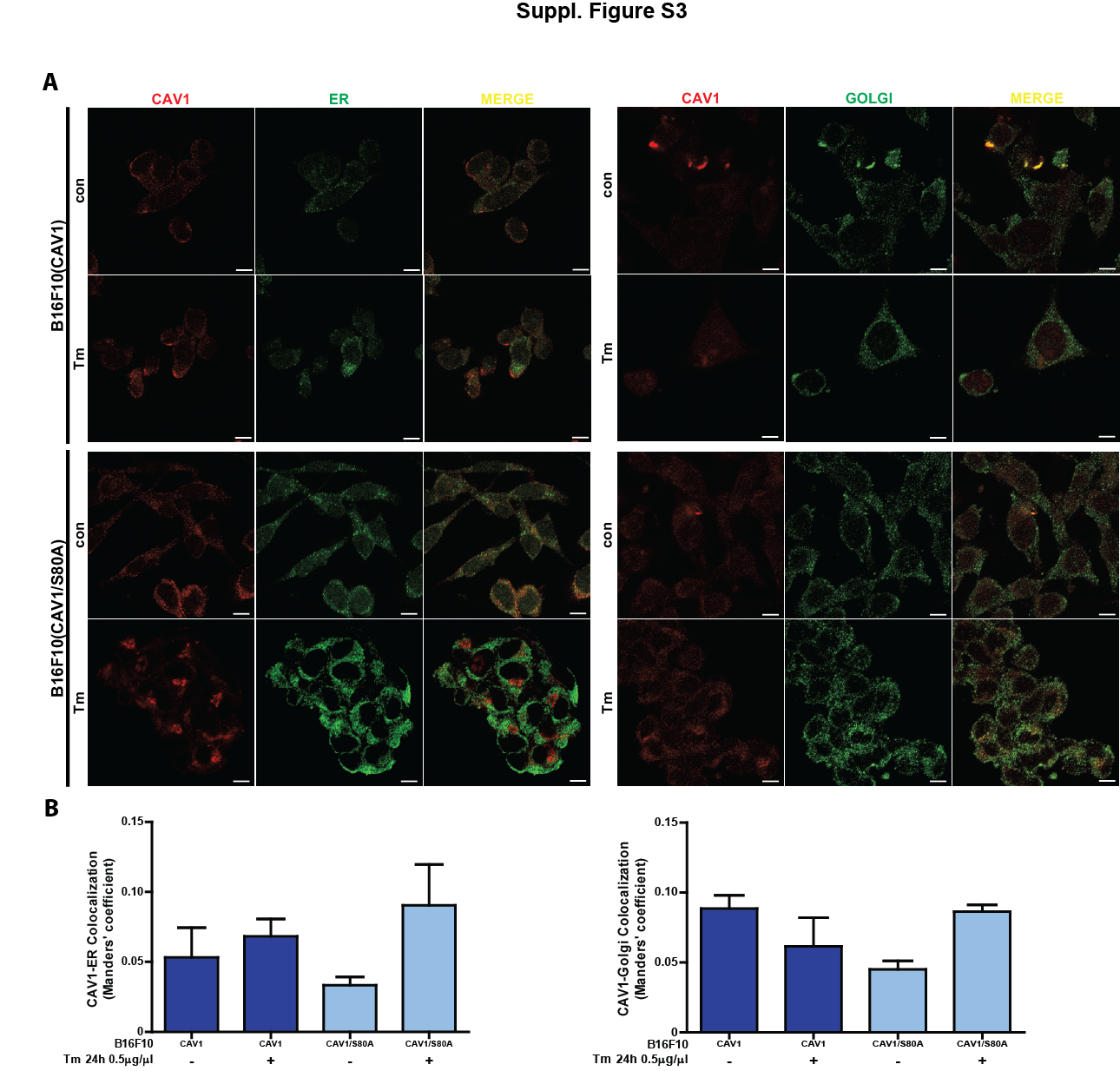

Supplement: Supplementary file 3 — Supplementary Figure [file 41419_2020_2792_MOESM3_ESM.png]

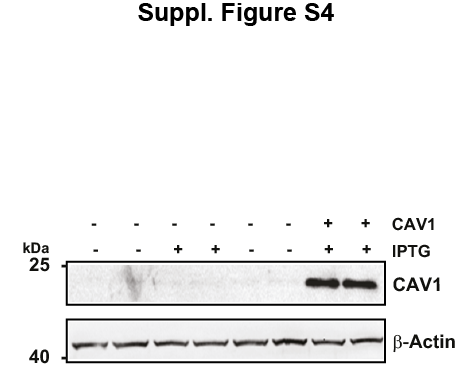

Supplement: Supplementary file 4 — Supplementary Figure [file 41419_2020_2792_MOESM4_ESM.png]

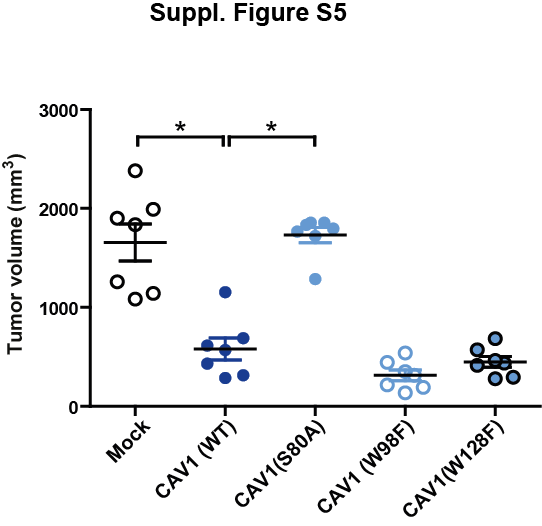

Supplement: Supplementary file 5 — Supplementary Figure [file 41419_2020_2792_MOESM5_ESM.png]

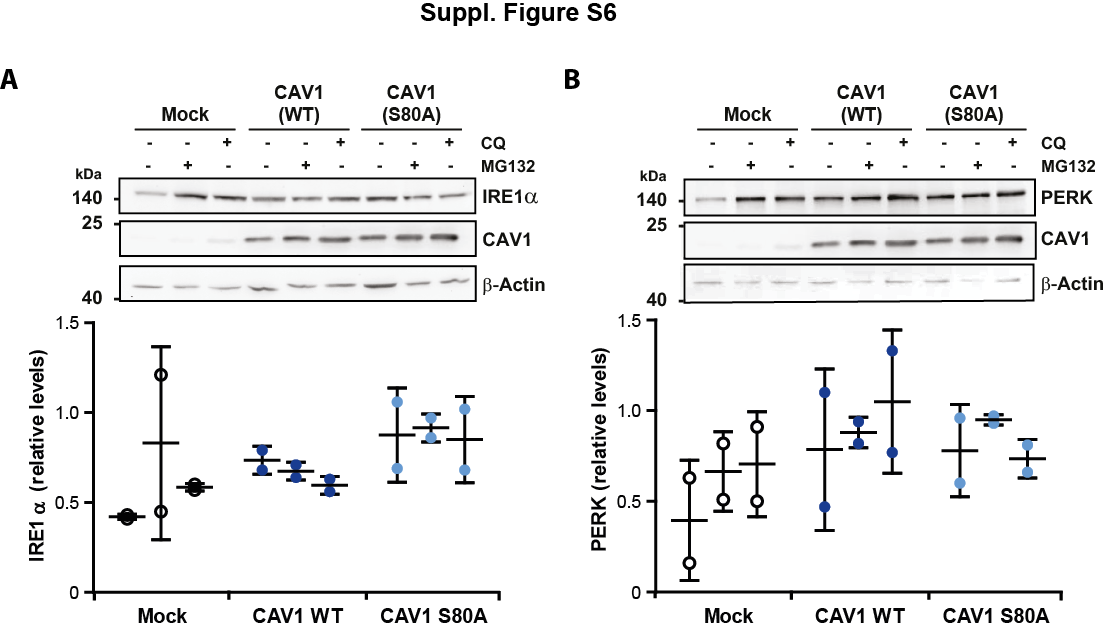

Supplement: Supplementary file 6 — Supplementary Figure [file 41419_2020_2792_MOESM6_ESM.png]
