# Supplementary material for: Caveolin-1 suppresses tumor formation through the inhibition of the unfolded protein response
Source: Cell Death Dis. 2020 Aug 3;11(8):648. doi: 10.1038/s41419-020-02792-4 (PMC7434918; doi:10.1038/s41419-020-02792-4)
Supplement: Supplementary file 7 — Supplementary Figure legends [file 41419_2020_2792_MOESM7_ESM.docx]

**Supplementary Figure Legends**

**Supplementary Fig S1. B16F10 and MDA-MB-231 cells do not express E-cadherin**

B16F10 cells were stably transfected either with empty vector (B16F10(Mock) or a vector containing the CAV1 insert (B16F10(CAV1). B16F10 cells were cultured for 48 h in the presence or absence of IPTG (1 mM). Total protein extracts were prepared for Western blotting and the expression of E-cadherin, CAV1 and β-actin were evaluated. In addition, endogenous CAV1 levels in the metastatic breast cancer cell line MDA-MB-231 were reduced using the specific short hairpin RNA construct shCAV1 and compared to control cells transduced with a control short hairpin construct (shCnt). Afterwards, cells were cultured for 48 h. Expression of E-cadherin, CAV1 and β-actin was assessed by Western blotting. Results shown are representative of n=2 independent experiments.

**Supplementary Fig S2. CAV1 is located to the plasma membrane and intracellularly in MDA-MB-231 cells**

A. MDA-MB-231 (wild-type) cells, expressing high endogenous levels of CAV1, were cultured for 48 h (control; con) or after 24h, treated with 0.5 μg/μL Tm for 24 h (Tm). Cells were fixed for immunofluorescence to evaluate the expression and localization of CAV1 and KDEL (marker of ER, n=4 experiments) or Golgin-97 (marker of Golgi; n= 3 experiments). Results shown are representative images. Arrows indicate areas where CAV1 staining is localized to the cell surface. Scale bar 10μm.

B. Images were analyzed to obtain Manders’ coefficients, as a measure of colocalization. CAV1-ER colocalization (n=4 images with approximately 10 cells per image, mean ± SEM), CAV1-Golgi colocalization (n=3 images with approximately 10 cells per image, mean ± SEM).

Our immunofluorescence analysis revealed that CAV1 was ubiquitously distributed in MDA-MB-231 cells under basal (control) conditions, localizing both to the cell surface (arrows) and the cytoplasm. Moreover, CAV1 colocalized to a limited extent with both ER and Golgi. Under ER stress conditions, Manders’ coefficient analysis suggests that there were no changes in the colocalization with these intracellular organelles. Therefore, our results imply that in MDA-MB-231 cells CAV1 is present at the plasma membrane and also associated with membranous intracellular compartments, specifically ER and Golgi.

**Supplementary Fig S3. CAV1 is heterogeneously located in B16F10 cells**

A. B16F10(CAV1) and B16F10(CAV1/S80A) were cultured for 48 h in the presence of IPTG (1 mM) (control; con) or after treatment with 0.5 μg/μL Tm for 24 h (Tm). Cells were fixed for immunofluorescence to evaluate the expression and localization of CAV1 and KDEL (marker of ER, n=4 experiments) or Golgin-97 (marker of Golgi; n= 3 experiments). Results shown are representative images. Scale bar 10μm.

B. Images were analyzed to obtain Manders’ coefficients, as a measure of colocalization. Histograms depict colocalization analysis for B16F10(CAV1) and B16F10(CAV1/S80A). CAV1-ER colocalization (n=4 images with approximately 10 cells per image, mean ± SEM), CAV1-Golgi colocalization (n=3 images with approximately 10 cells per image, mean ± SEM).

In B16F10(CAV1) cells, CAV1 localized to the cell surface and was detectable inside the cell. Under ER stress conditions, there was a modest increase in intracellular CAV1 localization in B16F10(CAV1) cells. However, Manders’ coefficient analysis suggests that CAV1-ER and CAV1-Golgi colocalization did not change under ER stress conditions in these cells. However, for CAV1(S80A) a significant increase in ER/Golgi localization was detected upon inducing ER stress.

**Supplementary Fig S4. IPTG-induced CAV1 expression in B16F10 ex-tumor cells in culture**

B16F10(Mock) and B16F10(CAV1) cells (300 000) were injected subcutaneously into C57BL/6 mice and tumor growth was monitored. After 15 days, mice were sacrificed, tumor tissue was trypsinized and isolated cells were cultured for two passages (ex-tumor cells). Total protein extracts were prepared from ex-tumor cells (Mock and CAV1) and analyzed by Western blotting for the expression of CAV1 in the absence or presence of IPTG (1 mM). β-actin was included as a loading control. Results shown are representative of n=2 independent experiments.

**Supplementary Fig S5. CAV1 tumor suppressor function is prevented by the S80A mutation**

B16F10(Mock), B16F10(CAV1), B16F10(CAV1/S80A), B16F10(CAV1/W98F) and B16F10(CAV1/W128F) cells were cultured for 48 h in the presence of IPTG (1 mM) to induce CAV1 expression. Then 300 000 cells were injected subcutaneously into C57BL/6 mice and tumor development was evaluated up to 15 days when mice were sacrificed. Tumor volumes measured for each group obtained at day 15 are shown (n=7, mean ± SEM, Kruskal-Wallis test, **p*<0.05).

For tumors in C57BL/6 mice evaluated 15 days after injection of cells expressing the mutant proteins, the S80A mutant was the only one where the tumor suppressor effect was lost and tumor volume was similar to B16F10(Mock) cells.

**Supplementary Fig S6. CAV1 expression does not promote IRE1α/PERK degradation**

A. B16F10(Mock), B16F10(CAV1) and B16F10(CAV1/S80A) were cultured for 48 h in the presence of IPTG (1 mM) and cells were treated with MG132 or chloroquine (CQ) for 12 h and total protein extracts were obtained for Western blotting to evaluate expression of UPR sensor IRE1α. β-actin was included as a loading control (n=2, mean ± SD).

B. B16F10(Mock), B16F10(CAV1) and B16F10(CAV1/S80A) were cultured for 48 h in the presence of IPTG (1 mM) and cells were treated with MG132 or chloroquine (CQ) for 12 h and total protein extracts were obtained for Western blotting to evaluate expression of UPR sensor PERK. β-actin was included as a loading control (n=2, mean ± SD).
